# Respiratory Syncytial Virus: Current Progress in Vaccine Development

**DOI:** 10.3390/v5020577

**Published:** 2013-02-05

**Authors:** Rajeev Rudraraju, Bart G. Jones, Robert Sealy, Sherri L. Surman, Julia L. Hurwitz

**Affiliations:** 1 Department of Infectious Diseases, St. Jude Children’s Research Hospital, 262 Danny Thomas Place, Memphis, TN 38105, USA; E-Mails: Rajeev.rudraraju@stjude.org (R.R.); bart.jones@stjude.org (B.J.); robert.sealy@stjude.org (R.S.); sherri.surman@stjude.org (S.S.); 2 Department of Microbiology, Immunology and Biochemistry, University of Tennessee Health Science Center, 858 Madison Avenue, Memphis, TN 38163, USA

**Keywords:** respiratory syncytial virus, candidate vaccines, protective immunity

## Abstract

Respiratory syncytial virus (RSV) is the etiological agent for a serious lower respiratory tract disease responsible for close to 200,000 annual deaths worldwide. The first infection is generally most severe, while re-infections usually associate with a milder disease. This observation and the finding that re-infection risks are inversely associated with neutralizing antibody titers suggest that immune responses generated toward a first RSV exposure can significantly reduce morbidity and mortality throughout life. For more than half a century, researchers have endeavored to design a vaccine for RSV that can mimic or improve upon natural protective immunity without adverse events. The virus is herein described together with the hurdles that must be overcome to develop a vaccine and some current vaccine development approaches.

## 1. RSV, the Virus

Respiratory syncytial virus is a negative strand RNA virus in the family *Paramyxoviridae*, the subfamily *Pneumovirinae* and the genus *Pneumovirus*. RSV was first discovered in 1952 as the cause of a serious lower respiratory tract disease, most pronounced among children in their first year of life [1]. Other individuals who are susceptible to severe RSV disease include patients with cardiac and pulmonary disorders, patients with immunodeficiencies, and the elderly [2,3,4,5,6]. RSV infection generally presents as an upper respiratory tract (URT) infection which progresses for several days before virus traffics to the lung [1,7]. Most infants are able to clear virus without extreme adverse events, but in the United States approximately 2% require hospitalization [8,9]. Globally, RSV infections are estimated to result in up to 199,000 deaths annually in children younger than 5 years of age [10] and treatment options remain controversial. Re-infections can occur, but generally result in milder disease; risks of infection are inversely associated with RSV-specific serum neutralizing antibody titers [11,12]. For infants at risk for first infections, the passive transfer of monoclonal RSV F-specific antibodies is recommended, but this type of prophylaxis is expensive and unavailable for most individuals who need it [13]. A licensed vaccine, the single best health care solution to infectious disease, is unavailable in the RSV field.

The RSV genome consists of 11 coding sequences including NS1, NS2, N, P, M, SH, G, F, M2-1, M2-2 and L [1,14]. The predominant proteins on the outer membrane of the mature virus are G, F and SH. Initiation of infection is usually mediated by the interaction between RSV G, the major attachment glycoprotein, and the host cell membrane. The viral receptors for G (and F) proteins on mammalian cells are not fully defined, but G can bind highly sulfated heparin-like glycosaminoglycans [15] as well as the CX3CR1 fracktalkine chemokine receptor [16]. G attachment facilitates F-mediated membrane fusion, although F protein can bind cell membranes independently (e.g., via binding to cellular heparan sulfate and nucleolin [16,17]) and mediate virus infection in the absence of G [1,18]. Fusion occurs when the F1 subunit of F protein is inserted into the target membrane and the protein refolds into a hairpin structure bringing virus and cell membranes into close proximity [19]. Upon membrane fusion, virus material including a single negative strand RNA genome is released into the cell cytoplasm [19]. Viral RNA is then transcribed and replicated yielding viral mRNA and new virus genome. New virions are assembled at the cell membrane, bud using cell membranes as their outer coat, and then target new cells for additional rounds of infection. Non-structural proteins support virus production while down-regulating host cell growth and defense mechanisms [1].

## 2. Hurdles to Vaccine Development

A main hurdle for RSV vaccine development was encountered in the 1960s during the testing of a formalin-inactivated RSV vaccine (FI-RSV). Unfortunately, the vaccinated infants were not protected from RSV infection. Instead, an unusually large percentage of the vaccinated children, when subsequently exposed to RSV by natural causes, developed disease severe enough to require hospitalization and there were two vaccine-related deaths [20]. It is essential that such a scenario never be repeated, and because the reason for the vaccine-related adverse events has not been confirmed, scientists are unsure precisely how to proceed. There remains no licensed vaccine product after one-half century of additional research.

The cause of vaccine-related deaths remains a point of discussion. One potential explanation is as follows: The formalin treatment of RSV altered membrane proteins on the virus and in so doing, rendered a vaccine that induced antibody responses that were non-neutralizing [21,22]. The inactivated vaccine also failed to elicit robust CD8+ T cells, because these classical killers of virus-infected targets are best induced by endogenously expressed viral antigens. In the absence of robust neutralizing antibodies and CD8+ T cells, RSV persisted and induced an aggressive CD4+ T cell and cytokine response in the lower respiratory tract [23,24]. Uncontrolled RSV replication and persistent inflammation led to blockage of the small airways in infants, leading to substantial morbidity and mortality [1,4,14,25].

There is now debate as to which immune cell populations or effector molecules were responsible for disease [21,26,27,28,29,30,31,32,33]. In a murine model designed to recapitulate the clinical outcome with FI-RSV, it was demonstrated that a subset of RSV G-specific CD4+ Th2 cells and eosinophils associated with disease. [23,24,31,32,34,35]. Additional mouse experiments showed that inhibition of IL-4 and IL-10 Th2 cytokines abrogated pulmonary histopatholgy [24]. Some researchers argue that the induction of Th2 cells, or perhaps the induction of any RSV-specific T cells should be avoided [36], even though T cells may be key providers of help for B cells and cytotoxic T lymphocyte functions at the time of RSV exposure. Others argue that the large majority of granulocytes described in original clinical autopsy reports were neutrophils rather than eosinophils [37], questioning the absolute comparison between mouse and human responses to FI-RSV. The potential benefits provided under the appropriate conditions by Th2 cells, other CD4+ and CD8+ T cell subsets, and eosinophil populations are contemplated [37,38,39,40,41,42]. For example, it is noted that individuals who lack T cells can experience great difficulty in RSV clearance and suffer worse outcomes than their immunocompetent counterparts [4,14,43,44]. It is further noted that eosinophils can be beneficial in that their transfer to the lungs of RSV-infected mice can enhance RSV clearance and inhibit airway hyper-reactivity [45]. Until debates are resolved, researchers and regulatory boards struggle to define 'go' and 'no go' criteria for the advancement of candidate vaccines, particularly when clinical studies target the pediatric arena. It is possible that past RSV vaccine candidates may have proved safe and efficacious in children, but were never tested in seronegative, pediatric populations. Perhaps a solution resides in comprehensive data analyses, revealing the complexities of the immune system and that each lymphocyte subset need not be categorically designated as beneficial or injurious [46]. Rather, as is the case in most RSV-experienced adults, a variety of adaptive and innate immune effectors including B cells, CD4+ T helper cells and CD8+ cytotoxic T lymphocytes function synergistically to mediate safe elimination of virus and virus-infected cells.

Another issue pertinent to RSV vaccine development concerns the selection of an appropriate human test population. Vaccine studies in young infants are performed with hesitancy due to the devastating experience with the FI-RSV vaccine. One suggested possibility is that vaccines might be tested in seropositive adults including the elderly rather than in infants. A number of strategies are considered including: (i) standard vaccination of placebo and control groups with subsequent assessment of disease caused by natural RSV exposure, (ii) the use of an experimental RSV challenge virus [47], and (iii) the vaccination of pregnant females to measure protection afforded to the infant at birth. One or more of these strategies may prove fruitful provided that attention is paid to a number of confounding variables. First, due to varying degrees of seropositivity in older individuals, immune responsiveness toward vaccination may be difficult to interpret. Individuals with high pre-existing immunity might be expected to show a boost in antibody titers following vaccination, but may instead clear vectors and antigens so rapidly that there is little opportunity for immune cells to re-activate. Second, vaccine safety data may be difficult to interpret due to frequent, unrelated disease complications in the oldest adults. One additional variable to be considered (upon design of either pediatric or adult vaccine protocols) is that individuals with poor diets or poor metabolism (e.g., vitamin deficiency) may exhibit general defects in immune responses toward respiratory virus vaccines [48,49]. With these considerations in mind, it has been proposed that RSV vaccine studies in the elderly are feasible, but would require the recruitment of thousands of participants to ensure fair vaccine assessment [50]. Another recent proposal has been that RSV vaccine studies might best be conducted not in the youngest infants or in adults, but in seronegative children who are at least 6 months of age [51]. Perhaps efficacy studies in older, seronegative children could assist vaccine licensure for a restricted age group while prompting additional clinical studies in the youngest infants.

## 3. Current RSV Vaccine Strategies

The first formal vaccine development effort occurred two centuries ago when Edward Jenner demonstrated that material from a cowpox lesion could serve as a vaccine for smallpox [52]. Successful vaccination, both then and now, relies on the safe introduction of a pathogen's antigenic determinants to the immune system. If the vaccine’s antigenic determinants are well matched to those of the native pathogen (as was the case for cowpox and smallpox), the antigens will activate pathogen-specific lymphocytes. In the case of virus-specific B cells, some effectors will mature to the plasma cell stage and constitutively secrete antibodies into blood, lymph and mucosal secretions, while others will maintain memory status, capable of immediate re-activation upon pathogen exposure. Activated CD8+ T cells are classically known for their killing of virus infected cells, while CD4+ T cells, (including Th1, Th2, Th17, TFh, Treg, and other subsets) are known for their support and regulation of CD8+ and B cell activities [53,54,55,56,57]. The heightened or “primed” state of immune surveillance may last months or years to inhibit pathogen entry and pathogen-mediated damage to the host [58]. Generally, vaccines are developed by: (i) inactivating the virus, (ii) identifying a related virus in another species that is safe in humans (the Jennerian approach), (iii) attenuating the virus, or (iv) using recombinant technology to present viral antigens, often in the context of a replication competent or replication-incompetent vector. In the RSV vaccine field, because of the outcome of the FI-RSV vaccine study, the first approach is generally discouraged, even if candidate vaccines retain RSV neutralizing determinants. The second strategy, the Jennerian approach, has not been advanced due to an insufficient antigenic match between bovine and human RSV [59]. The third and fourth strategies are the topics of most current vaccine research, described in greater detail below. Preclinical testing of vaccine candidates is generally accomplished first in small animals and then in non-human primates. Most small animal studies use hamsters, BALB/c mice, or cotton rats [60,61,62,63] while non-human primate studies generally use African green monkeys, rhesus macaques and chimpanzees [64,65,66]. Each animal is at least semi-permissive for RSV infection and is therefore advantageous in that immune responses and immunopathological events can be measured, but no animal model fully predicts the course of immune responses and disease in humans. In one instance, for example, an attenuated RSV vaccine appeared to be safe in non-human primates, but proved unsafe when tested in seronegative children [67]. Again, the debates described above concerning: (i) interpretation of data from animal experiments, and (ii) selection of clinical trial target populations, must be considered to determine when and how to advance vaccine candidates from pre-clinical to clinical trials.

### 3.1. Attenuated Virus Vaccines: Testing Variant Mutations

One strategy for RSV vaccine development has been to attenuate virus by cold adaption [68,69]. An early product of this research was cpts-248/404 [70,71]. However, in the youngest infants, the vaccine caused URT congestion associated with peak virus recovery and was deemed unacceptable for further development. More recently the cpts-248/404/1030/ΔSH vaccine was developed by introducing further mutations and deleting the SH gene, resulting in a more satisfactory product [72]. In a recent clinical study with this candidate, post-vaccination nasal washes revealed viruses with partial loss of the temperature sensitive phenotype, often due to a tyrosine/asparagine substitution in the L gene at position 1321 [73]. Researchers corrected the problem by creating a reversion-resistant virus with an alternative attenuating codon at position 1321. However, they then discovered a compensatory mutation at position 1313, forcing a deletion of that position (Δ1313) to yield a safer vaccine. When the Δ1313 deletion was paired with an NS2 gene deletion and tested at incrementally increasing temperatures, another compensatory mutation was discovered, I1314T. Finally, a vaccine with a new combination of mutations (ΔNS2/Δ1313/1314L) is being developed for evaluation in phase I clinical trials [74]. Reverse genetics, a powerful technology that allows the manipulation of viral genomes and recovery of infectious, recombinant virus particles, has assisted the progress described above [75].

A number of attenuated RSV vaccines have now been tested clinically. Currently, a phase I clinical study in adults, seropositive children and seronegative children is in progress to test safety and immunogenicity of an intranasally (I.N.) delivered RSV M2-2 deletion mutant [73,76,77].

The development of live-attenuated vaccines presents significant challenges, particularly when vaccines are delivered by the respiratory route to neonates. A concern is that viruses with compensatory mutations in the live-attenuated vaccines may associate with reversion to pathogenic phenotypes and lead to increased frequencies of adverse reactions *in vivo*. There is also a difficulty related to manufacturing and distribution, as RSV is naturally sensitive to changes in temperature, and attenuated strains by definition are difficult to propagate to high titers. 

### 3.2. Recombinant Protein Vaccines

Recombinant technology provides great flexibility both in terms of the RSV antigen(s) and the vector(s) with which the antigen is expressed. The major target antigens of recombinant vaccine technology are RSV G and F, as these are each capable of eliciting neutralizing antibodies as well as T cell responses. F is particularly attractive due to its considerable conservation among RSV isolates. Another antigen of recent interest is the small hydrophobic protein, SH [78].

As an example of progress in recombinant vaccine technology, Novartis is developing a postfusion RSV F trimer that elicits neutralizing antibodies and protection against RSV challenge in cotton rats [79]. Most researchers strive to match vaccine protein with pathogen protein to take advantage of polyclonal B and T cell responses that can work in unison to recognize and combat pathogen. Other researchers target particular epitopes such as a central conserved region of the G protein that induces antibodies to block the CX3C-CX3CR1 interaction. Mice vaccinated with fragments containing the CX3C motif have been shown to generate immune responses that can reduce lung virus titers and pulmonary inflammation following RSV challenge [80]. Still other researchers, as described above, propose that all T cell epitopes (and many B cell epitopes) should be removed from RSV vaccines [36,42]. Using computational design, epitope-scaffold vaccines have been developed to mimic an individual epitope on the F protein known to correspond with a neutralizing monoclonal antibody activity (motavizumab, [13]). In one case, several scaffolds were developed based on 13 discontinuous RSV F contact residues (xSxxLSxINDxxxxNDxKKLxSNx) for motavizumab. An automated search of protein structures supported the selection of three proteins as scaffolds: protein Z, a domain of protein A from *Staphylococcus aureus*, Cag-Z from *Helicobacter pylori* and the p26 capsid protein from equine infectious anemia virus. Amino acids outside of the motavizumab epitope were then modified or removed to optimize stability, solubility and motavizumab binding affinity. Critical to decisions concerning scaffold design are demonstrations that B cell and T cell determinants on viruses are often dependent on structural and spatial context, as regions outside the epitope affect 3-dimensional folding, post-translational modifications, and antigen processing [81,82,83,84]. For this reason, immune cells that respond to a protein fragment in one context (e.g., vaccine) do not necessarily recognize the same fragment when the context is changed (e.g., virus). When the *Stapylococcus aureus* protein A scaffold was fused to a pan-HLA DR binding epitope and tested for immunogenicity in mice, it induced RSV-binding antibodies, but these antibodies failed to neutralize RSV [36]. A separate study of a motavizumab-based scaffold in non-human primates illustrated neutralizing antibodies in a fraction of animals [85].

Most protein vaccines, whether designed to match unmanipulated viral proteins or targeted determinants, are combined with adjuvants. A plethora of adjuvants now exist including W_80_5EC [86], alum, 3-O-desacyl-4’-monophosphoryl lipid A (MPL), muramyl dipeptide (MDP), natural host defense peptides, CpG oligodeoxynucleotides (ODN) and polyphosphazenes. Polyphosphazenes are synthetic water-soluble polymers containing an inorganic backbone of alternating phosphorus and nitrogen atoms. Adjuvants are in some cases known to trigger cell molecules (e.g., toll-like receptors, TLR) to activate innate and adaptive immune responses. For example, MPL, CpG ODN, and MDP are ligands for TLR-4, TLR-9, and NOD2, respectively. The W_80_5EC product can serve both as an adjuvant and as a virus-inactivation method [86]. While adjuvant choices are many, U.S. Food and Drug Administration (FDA)-approved and licensed adjuvants are limited (alum and MPL). There is also a large variety of combinations for formulations of liposomes, nanoparticles or microparticles (synthetic particles and/or particles encompassing bacterial or viral components [87]) for the delivery of RSV proteins, peptides, and/or adjuvants. As an example, a truncated, secreted, trimeric F protein has been formulated for I.N. delivery with combinations of a TLR agonist (CpG ODN), an innate defense regulator peptide (IDR1002-VQRWLIVWRIRK), and polyphosphazene as nano- or microparticles, to induce RSV protective immunity [88,89]. Yet another example is Novavax's near-full length F glycoprotein formulated as a nanoparticle vaccine. This vaccine has been tested in healthy adults and has been shown to induce significant increases in the anti-F antibody response, including micro-neutralizing activities and competitive activity against the neutralizing monoclonal antibody Palivizumab [13,90,91,92]. An additional use of adjuvant has been with MPL combined not with a recombinant protein or particle, but with virosomes comprising membranes from RSV [93].

### 3.3 Replication Competent, Recombinant Viral Vaccines

Reverse genetics has assisted the development of recombinant viral vaccines that can serve as delivery systems for RSV antigens. One such vaccine, which has been well advanced in clinical trials, is MedImmune’s MEDI-534 [94,95]. This vaccine is a replication-competent vaccine that expresses RSV F [75,96]. The backbone is based on a bovine parainfluenza virus type 3 with substituted human PIV3 F and HN glycoproteins. MEDI-534 was tested in non-human primates and also in a phase I study in young children between the ages of 6 months and <24 months. Doses of 10,000, 100,000 and 1,000,000 TCID_50_ were tested in the clinical trial and RSV specific antibody responses were noted in 50% of vaccinees administered three 1,000,000 TCID_50_ doses of vaccine with two month intervals. Virus that was shed from study participants revealed genetic changes that were associated with reduced RSV F protein expression. A close analysis of the MEDI-534 vaccine then demonstrated that some of the same genetic variants were minor components of the administered vaccine [97]. The implication of these sequence variants is a current topic of discussion.

Another promising candidate is St. Jude’s recombinant, replication competent vaccine (SeVRSV), also developed with reverse genetics technology [75]. Sendai virus (SeV), a mouse parainfluenza virus type I with a high sequence and antigenic similarity to human parainfluenza virus type I (hPIV-1), was used as the vaccine’s backbone [98,99,100,101,102]. SeV is an attractive vaccine candidate and vaccine backbone, because there has never been a confirmed case of SeV-associated disease in humans. The species specificity of Sendai virus is attributed in part to its unique sensitivity to human type I interferon [103]. In small animals a single I.N. dose of SeV induced B and T cell responses within days after immunization that lasted for the animal's lifetime without need for a booster [62]. The SeVRSV recombinant carries the RSV F gene and thereby instructs its expression in infected cells [104]. When tested in cotton rats, SeVRSV protected animals from challenge with both A and B RSV isolates. SeVRSV could also be mixed with two additional SeV-based vaccines in a single I.N. inoculation to protect against four different challenge viruses: RSV, hPIV-1, hPIV-2 and hPIV-3 [62]. When SeVRSV was tested as a vaccine in African green monkeys with non-recombinant SeV as a control, it safely and fully prevented infection of the lower airways following RSV challenge [66]. The non-recombinant SeV has already entered clinical trials as a hPIV-1 vaccine and has been well tolerated in adults [105] and 3–6 year old children. SeVRSV is now being manufactured for testing in an age de-escalation clinical trial. Previous pre-clinical and clinical data suggest that SeVRSV will safely protect children from both RSV and hPIV-1 infections. When compared to the live-attenuated RSV vaccines, SeV based vaccines benefit from their relative stability to temperature changes and ease of growth to high titers in chicken eggs and cell cultures, a boon for manufacturing and vaccine distribution.

### 3.4 A Variety of new Vectors and Concepts in the RSV Vaccine Field

A great number of additional recombinant vaccines are in stages of pre-clinical testing. Vectors include Semliki Forest virus [106], Venezuelan equine encephalitis virus [107,108], adenovirus from humans or non-human primates [109,110], influenza virus [111], measles virus [112], Newcastle disease virus like particles (VLPs [113]) and plasmid DNA [114,115]. Other vaccine delivery systems are based not on viruses, but bacteria, yeast or plants [116], such as the Mucosis SynGEM® vaccine, an I.N. vaccine that presents native trimeric F protein formulated in a non-living bacterium-like particle (BLP) [117]. There have also been combination prime-boost strategies using one form of recombinant vaccine followed by another. For example a recombinant vaccine prime based on replication-defective chimpanzee adenovirus can be followed with a boost based on modified vaccinia Ankara (MVA) [118], or a recombinant DNA prime can be followed by recombinant adenoviral vector boosts [119]. This article describes a portion, but not all of the vaccine candidates that are currently under investigation. Table 1 provides a short list as a sampling of vaccine strategies with references and review articles. It is quite likely that one or more than one of the current vaccine candidates will prove successful. The advanced development and licensure of a safe and effective RSV vaccine will indeed be momentous, a long-awaited milestone for the prevention of the significant sickness and death caused by RSV infections.

**Table 1:** Sample respiratory syncytial virus (RSV) vaccine references and review articles.

Vaccine Type	Sample references
Attenuated RSV	[73,74]
Inactivated RSV	[86,120]
RSV protein(s) adjuvanted and/or as micro/nano-particles	[79,89]
Epitope scaffold	[36]
Virosome	[93,121]
Virus like particle (VLP)	[113,122]
Replication competent virus-based vector	[66,94,95,97]
Bacteria-based vector	[123,124,125]
Plant-based vector	[116]
Prime-boost with heterologous vectors	[118,119].
**Related review articles**	**[1,4,14,25,115,126]**
